# Characteristics and Outcomes of Intensive Care Unit Survivors: Experience of a Multidisciplinary Outpatient Clinic in a Teaching Hospital

**DOI:** 10.6061/clinics/2017(12)08

**Published:** 2017-12

**Authors:** Péricles A.D. Duarte, Jaquilene Barreto Costa, Silvana Trilo Duarte, Sheila Taba, Claudia Regina Felicetti Lordani, Erica Fernanda Osaku, Claudia Rejane Lima Macedo Costa, Dalas Cristina Miglioranza, Daniela Prochnow Gund, Amaury Cesar Jorge

**Affiliations:** I Unidade de Terapia Intensiva Geral, Hospital Universitario do Oeste do Parana, Cascavel, PR, BR; II Departamento de Psicologia, Hospital Universitario do Oeste do Parana, Cascavel, PR, BR; III Departamento de Fonoaudiologia, Hospital Universitario do Oeste do Parana, Cascavel, PR, BR; IV Departamento de Nutricao Clinica, Hospital Universitario do Oeste do Parana, Cascavel, PR, BR; V Departamento de Fisioterapia, Hospital Universitario do Oeste do Parana, Cascavel, PR, BR; VI Departamento de Servico Social, Hospital Universitario do Oeste do Parana, Cascavel, PR, BR

**Keywords:** Critical Care, Epidemiology, Rehabilitation, Quality of Life

## Abstract

**OBJECTIVES:**

To describe the experience of an outpatient clinic with the multidisciplinary evaluation of intensive care unit survivors and to analyze their social, psychological, and physical characteristics in a low-income population and a developing country.

**METHODS:**

Retrospective cohort study. Adult survivors from a general intensive care unit were evaluated three months after discharge in a post-intensive care unit outpatient multidisciplinary clinic over a period of 6 years (2008-2014) in a University Hospital in southern Brazil.

**RESULTS:**

A total of 688 out of 1945 intensive care unit survivors received care at the clinic. Of these, 45.2% had psychological disorders (particularly depression), 49.0% had respiratory impairments (abnormal spirometry), and 24.6% had moderate to intense dyspnea during daily life activities. Patients experienced weight loss during hospitalization (mean=11.7%) but good recovery after discharge (mean gain=9.1%), and 94.6% were receiving nutrition orally. One-third of patients showed a reduction of peripheral muscular strength, and 5.7% had moderate to severe tetraparesis or tetraplegia. There was a significant impairment in quality of life (SF-36), particularly in the physical and emotional aspects and in functional capacity. The economic impacts on the affected families, which were mostly low-income families, were considerable. Most patients did not have full access to rehabilitation services, even though half of the families were receiving financial support from the government.

**CONCLUSIONS:**

A significant number of intensive care unit survivors evaluated 3 months after discharge had psychological, respiratory, motor, and socioeconomic problems; these findings highlight that strategies aimed to assist critically ill patients should be extended to the post-hospitalization period and that this problem is particularly important in low-income populations.

## INTRODUCTION

As the survival rates of patients admitted to intensive care units (ICUs) improve, concerns have arisen regarding mid- to long-term morbidity in this population. Patients who survive a critical illness and hospitalization in an ICU (ICU survivors) may develop physical and/or psychological complications during hospitalization and after discharge from the ICU 1,2. In post-ICU follow-ups, psychological complications (such as depression and post-traumatic stress disorder [PTSD]), as well as motor (i.e., post-critical illness tetraparesis) and respiratory complications (i.e., restrictive and obstructive disorders) have been reported 3-5.

Still, the number of studies that have focused on the social and economic impacts of acute illness or on admission to an ICU is low 6. These topics are particularly important when considering the high intrinsic costs of an ICU admission and the wide variety of locations and countries that face the problem of post-ICU complications. Specifically, despite recent studies that have evaluated different characteristics of post-ICU patients 7-9, the economic and social impacts have been poorly assessed and described in low-income and developing countries, and the data are scarce. Most of the data used by the field was published by services in countries with higher economic development. Therefore, dissemination of an ICU survivor follow-up policy for low-income countries is extremely important in order to manage the quality of hospital assistance, monitor health rehabilitation, and plan costs and healthcare programs in these places.

This study aimed to describe the experience of an outpatient clinic that performed follow-up assessments of a general adult population of ICU survivors in a teaching hospital in a southern Brazilian city, analyzing the patients’ demographic, clinical, and socioeconomic profiles, together with the current clinical conditions of these survivors.

## METHODS

This cohort observational study investigated general adult ICU survivors in a teaching hospital in the western region of the state of Paraná (southern Brazil) from December 2007 to December 2014. Western Paraná University Hospital has 190 beds and is the tertiary reference center for 26 nearby municipalities (approximately 1.5 million inhabitants). The general ICU is a mixed unit (medical, surgical, and trauma). The ICU had 9 beds until 2012, when it was expanded to 14 beds.

Both the hospital and outpatient clinical records were analyzed to include patients admitted to the general adult ICU from December 2007 to December 2014 who were discharged alive from the hospital and evaluated at the Post-ICU Outpatient Clinic 3 months later. (Thus, the patients were evaluated at the Post-ICU Outpatient clinic between February 2008 and February 2015).

The data were tabulated in a Microsoft Excel spreadsheet containing data from the hospital and ICU stay, social and demographic characteristics, and from the consultations with the different professionals in the Post-ICU Outpatient Clinic.

### Post-ICU Outpatient Clinic

The Post-ICU Outpatient Clinic has a multidisciplinary team comprising a physician, nurse, physiotherapist, nutritionist, psychologist, speech therapist, and social worker. All professionals who work in the outpatient clinic are also members of the general ICU team.

Three months after discharge from the ICU, the patients were invited by telephone to come to the outpatient clinic, where they were seen by each professional on the team in a single session. Questionnaires and tests from each specialty were administered and lasted approximately 30 minutes for each professional. Patients who failed to attend were actively followed up by telephone, and a new date was scheduled. Patients who failed to appear after two attempts were excluded. Each patient was evaluated only once at the clinic, with no follow-up after this consultation. When necessary, the patient was referred to medical follow-up. The Post-ICU Outpatient clinic routines described herein were implemented and followed at the outpatient clinic independently of the current study. In this article, we only describe the results found during the study period.

### Inclusion and exclusion criteria

All patients hospitalized during the study period were included if they met the following criteria: stayed more than 24 hours in the ICU, were 18 years of age or older, were already discharged (and were therefore at home), and attended the outpatient evaluation. Once the patient attended the consultation at the clinic, no exclusion criteria were applied. However, in practice, not all evaluations could be conducted for each patient depended on the patient’s condition. For example, the psychological interview could not be performed with non-verbal patients or patients who had significant post-ICU neurological sequelae (detected during the outpatient consultation). For this reason, the number of patients per evaluation varies in the descriptions of the results.

### Protocols, criteria, and instruments

During the ICU stay, the patients were treated according to the protocols of the ICU regarding mechanical ventilation and weaning, sedation, nutrition and management of specific conditions such as sepsis and trauma.

Acute respiratory distress syndrome (ARDS) was identified retrospectively in accordance with the Berlin Definition 10. Sepsis was defined according to the ACCP/SCCM Consensus 11 (available at the time of the study). Trauma was defined as any acute condition caused by an external agent and included traffic accidents, violence (e.g., beatings, injuries from firearms or non-firearm weapons), falls, and workplace accidents, among others. The diagnostic definition of each acute or chronic disease (e.g., Chronic Obstructive Pulmonary Disease [COPD], Chronic Heart Failure [CHF]) was in accordance with the clinical diagnosis of the assisting medical team.

The Hospital Anxiety and Depression Scale (HADS) 12 was used to assess psychological complications in the outpatient evaluation, and the Impact of Event Scale-Revised (IES-R) was used to assess PTSD symptoms 13; the cut-off scores were ≥9 and >20, respectively.

The nutritional evaluation was performed by measuring the weight and height of the patient and reviewing the measurements or estimates of the weight made during hospitalization and discharge. The current form of nutrition (i.e., oral, enteral or gastrostomy) was recorded.

The physiotherapeutic evaluation comprised an analysis of respiratory and motor functions. Participants completed a questionnaire regarding dyspnea during different activities of daily living using a modified Borg scale 14. To assess respiratory muscle strength, a manovacuometer was used to measure the maximum inspiratory pressure (MaxIP) and maximum expiratory pressure (MaxEP). Muscle strength was evaluated by clinical examination using the Medical Research Council scale 15. Spirometry tests were performed using a One Flow Soft 1.2^®^ device (Essex, UK). When possible (because many patients were wheelchair-bound or bedridden), patients underwent a 6-minute walk test, and dyspnea evaluations and pulse oximetry were subsequently performed.

Social and economic conditions were assessed by the social worker using a questionnaire that assessed family income, the number of residents in the home, labor status, and financial support from the government.

The psychologist evaluated the quality of life with a questionnaire using the Short-Form Health survey (SF-36) scale 16. The mean value for each dimension was considered.

### Statistical analysis

Descriptive statistical analysis was performed, and the percentages were compared using the chi-square test. The occurrence of baseline characteristics was expressed as the frequency, mean, and standard deviation. The analyses of the baseline and epidemiological data and outcome was conducted using Student’s t-test, analysis of variance, and the Tukey test, assuming a significance level of *p*<0.05.

This study was conducted in accordance with the recommendations of Resolution 466/2012 of the Brazilian National Health Council. The project was approved by Western Paraná State University’s Permanent Committee on Ethics and Research involving human beings. Accordingly, post-informed consent was waived since the current study only describes the results of a population that had already previously been treated.

## RESULTS

**Figure 1:**
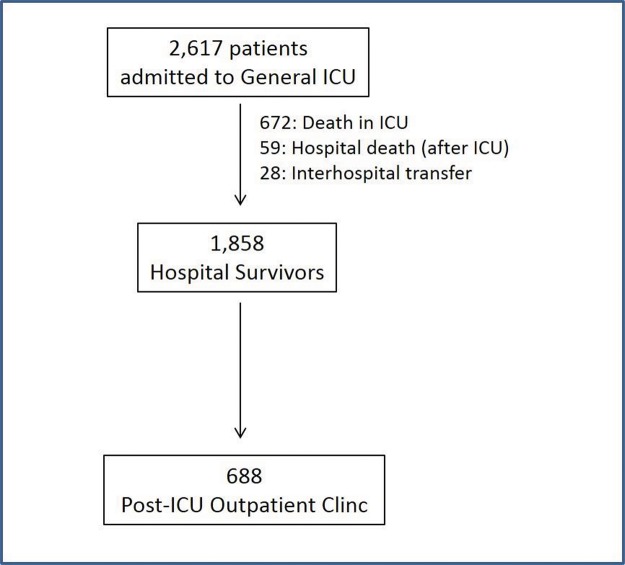
Patient flow. ICU: Intensive Care Unit.

During the study period, 2617 patients were admitted to the general ICU, and 1945 (74.3%) survived. Patients who were discharged alive from the ICU were transferred to the hospital ward. Of these patients, 688 were evaluated in the Post-ICU Outpatient Clinic (Figure 1). The most common reasons given for not attending the scheduled consultation (non-tabulated data) were a) transportation difficulties since a considerable number of patients lived in other municipalities; b) difficulty arranging for a family member to transport the patient to the consultation, especially in cases of elderly patients; and c) the patient’s clinical condition and limitations (e.g., a bedridden status or difficulty walking). The clinical and epidemiological characteristics of the patients admitted to the ICU, the survivors, and the patients seen at the Outpatient Clinic are presented in Table 1.

As expected, differences were detected between the patients admitted to the ICU and the survivors regarding the following: age, reason for hospitalization (medical patients had higher hospital mortality than non-medical patients), admission Acute Physiology and Chronic Health Evaluation (APACHE) II score, comorbid conditions (particularly COPD), and the use of invasive mechanical ventilation (MV) (see Appendix; data are not related to post-ICU patients and only related to ICU patients).

In contrast, when comparing the survivors and patients seen at the outpatient clinic, only differences in the presence of comorbid conditions were observed (Table 1).

At the outpatient evaluation, 45.2% of the patients exhibited a psychological alteration, including depression, anxiety, and PTSD symptoms. More than one problem was detected in nearly half (46.8%) of these patients. Most patients (84.4%) described having some memory of the events in the ICU. Of this group, 39.1% recalled memories of real events, and 45.3% had illusion memories (alone or in combination with real facts), such as dreams (13.3%), nightmares (7.0%), and hallucinations (25.0%).

Regarding the nutritional evaluation, nearly all participants (94.6%) (Table 3) were eating by mouth at the time of the outpatient evaluation, even though most received enteral nutrition during their hospital stay. The group had an 11.7% mean weight loss during the hospitalization period, with a 9.1% gain between hospital discharge and the outpatient consultation.

Motor impairment was common: 31.8% of the patients (Table 2) had a moderate or intense reduction in the strength of their extremities, and 5.7% had moderate or severe tetraparesis or tetraplegia.

Respiratory impairment was even more common than the motor impairment (Table 2). Half of the patients (49.0%) had abnormal spirometry tests. The most common finding was obstructive impairment. However, diaphragm muscle strength (estimated by MaxIP) was adequate: the median result was 70.0 mmHg, and only 14.1% exhibited MaxIP<40 mmHg. Dyspnea while performing routine daily activities (such as walking, cooking, or showering) was either mild or absent for 58.5% of the participants, and only 9.3% had intense dyspnea during physical activities.

**Figure 2:**
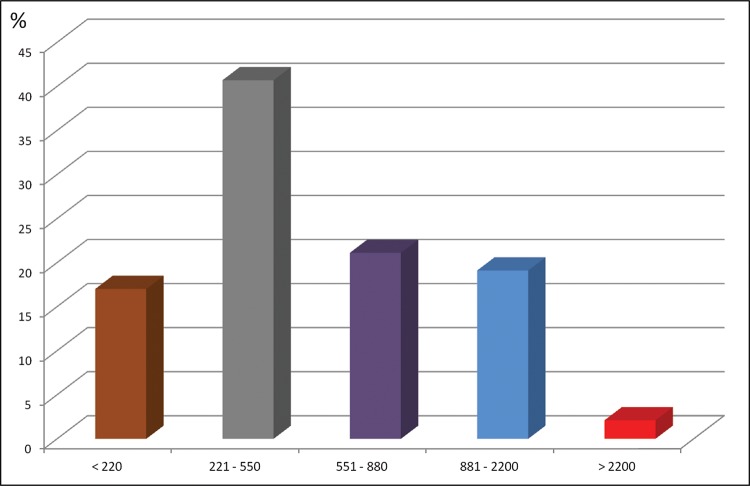
Monthly family income (US$).

Most patients were in precarious financial and social situations: over half had a total family income that was less than US$550.00 per month (Figure 2). Furthermore, half of the families (53.4%) were receiving some kind of financial support from the government (either at the federal, state, or municipal level) at the time of the outpatient evaluation. Among the 211 patients evaluated, 44 (20.8%) were retired or pensioners, 16 (7.6%) were unemployed, 13 (6.2%) were students, and 138 (65.4%) were formal or informal workers (13.8% of whom had returned to work).

**Figure 3:**
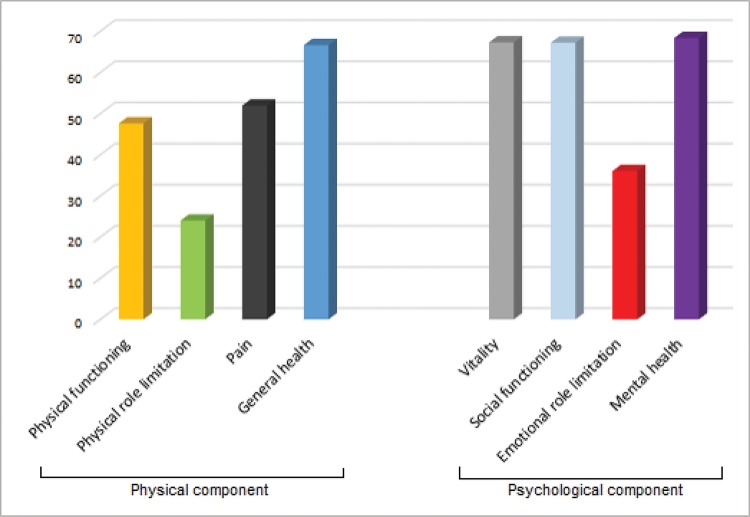
Quality-of-Life scores, according to SF-36 (scored from 0 to 100; the higher the value, the lower the disability; n = 322). SF-36: Medical Outcomes Study 36-Item Short-Form Health Survey.

The SF-36 questionnaire evaluation (Figure 3) showed a significant impairment in quality of life related to health; the mean score was low in all evaluated domains. The most affected items were physical functioning, physical role limitation, pain, and emotional role limitation.

## DISCUSSION

Despite the variety of factors associated with admission to an ICU (particularly the different kinds of diseases and conditions that affect this patient population), multiple mid- and long-term complications can clearly occur among ICU survivors. Such complications are proportional to the severity of the critical illness (e.g., organ failure), comorbidities and factors related to treatment in the ICU 17-19.

Mental disorders such as anxiety, depression, and PTSD may appear after hospitalization in an ICU. In our study, one of these disorders was detected in nearly half of the patients, and more importantly, almost 25% of the patients had more than one disorder. Previous studies have reported a prevalence of post-discharge anxiety ranging from 11.9% 20 to 44% 21, and the rates of post-discharge depression vary from 9.7% 20 to 30% 22. These studies also show that 14% to 27% of patients may develop PTSD 22-24. In patients admitted to an ICU, the highest rates of psychological complications (anxiety and depression) were associated with unfavorable socioeconomic conditions 25 or serious complications during ICU, such as ARF requiring dialysis 7. An important consideration is that, in our study, we have evaluated the presence of PTSD symptoms, which may have led to an overestimation of the incidence of this disorder 24.

Another important finding was the report of memory illusions (including hallucinations) by the surviving patients. The incidence of memory illusions may be correlated with psychological disorders and a reduction in quality of life among ICU survivors 26. Even among the “real” memories, 67.2% of the patients mentioned memories of confusion, agitation, physical restraint (53.7%), thirst (51.2%), and procedures (24.7%), such as the presence of a tracheal tube, tracheal aspiration, and extubation. Therefore, strategies for humanizing ICU care will have both short-term and long-term impacts on survivors.

In our experience of the follow-up outpatient clinic with a multidisciplinary team, we observed psychological and physical complications 3 months after discharge as well as the need to refer patients to other specialties. This finding confirms the need for follow-up outpatient clinics; however, few services follow their patients after discharge from the ICU, and the number of rehabilitation services that focus on patient recovery is small. A Brazilian study has shown that up to 40% of ICU survivors require rehospitalization during the first year post-ICU 18. Consequently, the concept of critical patient care expands to a more encompassing care perspective, and intensive care is considered a continuum that should also include patient follow-up after discharge.

Our respiratory evaluation results indicate that most of the patients did not present a significant gas exchange compromise and had adequate respiratory muscle strength. In addition, only a minority of the patients had moderate/severe dyspnea. The literature shows that the degree of respiratory impairment in survivors depends on the primary etiology of respiratory failure in the ICU. Despite the functional impairment, dyspnea and diminished quality of life of surviving patients with ARDS, most studies have shown that the residual impairment of gas exchange in these patients is small and that few survivors still exhibit hypoxemia or a need for domiciliary oxygen 4,28. In contrast, up to 70% of patients admitted to the ICU due to decompensated COPD required home oxygen therapy after discharge from the hospital 27, and the MaxIP of hospitalization survivors in this group is correlated with functional recovery and pulmonary rehabilitation 29. The authors believe that the relatively reduced respiratory impairment of our population can be justified mainly by the low prevalence of chronic respiratory diseases and the high incidence of young people with trauma (with adequate respiratory recovery).

Due to various factors, including serious injury, metabolic stress, inflammatory polyneuropathy, and nutritional disorders, critical patients suffer rapid deteriorations in adipose and muscular tissue mass 30-32. As a result, persistent muscular weakness is common during the first months after a critical illness, having a profound impact on respiratory and motor function and on quality of life 3,19,33. The relatively low incidence of severe tetraparesis/motor sequelae in our population may be due to the lower incidence of sepsis upon admission (which is the main factor related to residual muscle weakness) 3,19. However, another factor may be that patients with higher impairment (bedridden) did not attend the clinic, resulting in a bias in the incidence of severe tetraparesis/motor sequelae.

Nutritional conditions and weight variations are among the major immediate and late consequences of critical illness 34. Several related factors include dysphagia 1, loss of appetite 34, metabolic consumption of muscle mass, and loss of muscle mass due to inactivity 3. In the present study, significant weight loss was detected during hospitalization. However, considerable weight gain was also detected in the studied group during the post-hospitalization period. These findings highlight the importance of nutritional and speech therapeutic interventions in the rehabilitation of critical patients, particularly those with neurological impairments, since recovering the ability to eat by mouth is a goal that should be quickly attained.

Since this population is affected by psychological and physical disorders, the quality of life is compromised, particularly regarding physical components. A marked reduction in the quality of life has been demonstrated in post-ICU patients, particularly in the first months after discharge, with variable improvement after 6–12 months 35. A limitation of our quality-of-life analysis is our lack of pre-ICU data, which would further support the results of this study. However, since a significant percentage of patients (e.g., trauma patients) had no previous illnesses, the numbers are notable.

Even though our University Hospital is located in a city within Brazil’s wealthiest region (the south), our study population comprised low-income patients (treated in a public hospital) in a country with notorious shortcomings in its public healthcare system (despite important improvements in recent years, such as the creation of the Unified Health System, or SUS) 36. Very few hospitals in Brazil are focused on rehabilitation, and few cities have public home care services 37. Therefore, the vast majority of patients, even those with serious functional sequelae, must remain in their own homes, with either precarious specialized care (physiotherapy, nursing, nutrition, speech therapy) or none at all. This scene becomes even more problematic when considering that most patients in this study were victims of trauma, were young, and could therefore potentially return to their usual living conditions if only the treatment received at the hospital had been maintained during their post-hospital rehabilitation. Consequently, we must emphasize the social roles that the ICU teams play in learning about the realities of the population they serve and about the impacts of critical illness and hospitalization, particularly in low-income countries.

This study has several limitations, some of which are inherent to its nature. First, this was a single-center observational study. Therefore, any generalization of the described findings may be inadequate.

Another limitation refers to the number of patients attended in the outpatient clinic compared to the total number of patients discharged alive from the hospital. Social and medical factors may have prevented many families from transporting patients to the outpatient clinic. Thus, the data for the treated patients may not accurately reflect the clinical severity and precarious socioeconomic conditions of our population. However, the results obtained in our clinic have assisted efforts by our institution to improve post-hospital care (including social assistance).

Additionally, our outpatient clinic conducted only a single evaluation of the ICU survivors 3 months after discharge, so the long-term consequences in this population remain unknown. Currently, our Post-ICU Outpatient clinic is expanding the follow-up to 6 months after discharge. (Therefore, patients will be seen 2 times after leaving the ICU). In the future, we hope to describe this improved follow-up evaluation. Because this study was an observational study (applied to an already existing reality in ICU and post-ICU outpatient care), some relevant data could not be adequately analyzed or collected (e.g., correlations between post-ICU psychological disorders and the sedation time or type, motor characteristics with curare use, or nutritional status variables during the ICU stay) due to difficulties in accessing these data from the patients' charts. However, the execution of this study has provided the staff with several elements that can be used toward improving the collection of these data for possible future analyses.

The patient population in our study also had specific characteristics, including a high incidence of trauma, young age, and low-income status, and may not correspond to the populations of other ICUs or social realities.

A significant percentage of ICU survivors seen 3 months after discharge presented with psychological, respiratory, motor, and socioeconomic complications, emphasizing that strategies for the care of critically ill patients must be extended to the post-hospitalization period.

## AUTHOR CONTRIBUTIONS

Duarte PA designed the study, analyzed the data and wrote the manuscript. Costa JB, Duarte ST, Taba S, Lordani CR, Osaku EF, Costa CR, Miglioranza DC, Gund DP and Jorge AC collected the data, analyzed the data, and wrote the manuscript. All authors have read and approved the final version of the manuscript.

## Figures and Tables

**Table 1:** Patient demographic and clinical characteristics.

	ICU Total	ICU Survivors	Not attending the Outpatient Clinic	Attending the Outpatient Clinic	*p*-value Survivors (attending *vs*. not attending the Outpatient Clinic)
n	2617	1945	1257	688	- - - -
Male (%)	1639 (62.6%)	1227 (63.1%)	801 (63,7%)	433 (62.9%)	0.962
Age, years, n (%)	- - - -	- - - -		- - - -	- - - -
<40	1081 (41.3%)	897 (46.1%)	563 (44.8%)	335 (48.7%)	0.075
41-60	803 (30.7%)	567 (29.2%)	347 (27.6%)	212 (30.8%)	
61-75	524 (20.0%)	350 (18.0%)	249 (19.8%)	109 (15.8%)	
>75	209 (8.0%)	131 (6.7%)	98 (7.8%)	32 (4.7%)	
Reason for hospitalization, n (%)	- - -	- - -		- - -	- - - -
Trauma	963 (36.8%)	761 (39.1%)	478 (38.1%)	290 (42.1%)	0.134
Medical	916 (35.0%)	570 (29.3%)	386 (30.7%)	180 (26.2%)	
Elective surgery	429 (16.4%)	393 (20.2%)	257 (20.4%)	136 (19.8%)	
Non-traumatic urgent surgery	309 (11.8%)	221 (11.4%)	136 (10.8%)	82 (11.9%)	
APACHE II, n (%)	- - - -	- - - -		- - - -	- - - -
<5	39 (1.5%)	38 (2.0%)	24 (1.9%)	14 (1.9%)	0.129
6-10	188 (7.2%)	174 (8.9%)	115 (9.1%)	65 (9.5%)	
11-20	882 (33.7%)	788 (40.5%)	525 (41.8%)	260 (37.8%)	
21-30	1055 (40.3%)	760 (39.1%)	465 (37.0%)	292 (42.5%)	
>30	453 (17.3%)	185 (9.5%)	128 (10.2%)	57 (8.3%)	
Comorbid conditions, n (%)	- - -	- - -		- - -	- - - -
One or more comorbid condition	1633 (62.4%)	1157 (59.5%)	701 (55.8%)	456 (66.3%)	0.002
AIDS	49 (1.9%)	32 (1.6%)	24 (1.9%)	07 (1.0%)	0.343
Recent/current cancer	248 (10.7%)	225 (11.6%)	149 (11.8%)	76 (11.0%)	0.722
DM	237 (9.1%)	175 (9.0%)	97 (7.7%)	78 (11.3%)	0.092
Illicit drugs	64 (2.4%)	54 (2.8%)	28 (2.2%)	26 (3.8%)	0.238
COPD	175 (6.7%)	87 (4.5%)	54 (4.3%)	33 (4.8%)	0.828
Alcoholism	243 (9.3%)	210 (10.8%)	92 (7.3%)	118 (17.1%)	<0.001
SH	589 (22.5%)	486 (25.0%)	282 (22.4%)	204 (29.6%)	0.021
CHF	162 (6.2%)	126 (6.5%)	88 (7.0%)	38 (5.5%)	0.401
CRF	61 (2.3%)	41 (2.1%)	34 (2.7%)	07 (1.0%)	0.091
Moderate/severe obesity	170 (6.5%)	103 (5.3%)	56 (4.4%)	47 (6.8%)	0.173
Use of MV, n (%)	2269 (86.7%)	1610 (82.8%)	1037 (82.5%)	573 (83.3%)	0.810
Time on MV, days, n (%)	- - -	- - -		- - -	- - -
0	348 (13.3%)	335 (17.2%)	220 (17.5%)	115 (16.7%)	0.493
1-5	1112 (42.5%)	811 (41.7%)	513 (40.8%)	298 (43.3%)	
6-10	487 (18.6%)	335 (17.2%)	218 (17.3%)	117 (17.0%)	
>10	670 (25.6%)	464 (23.9%)	306 (24.4%)	158 (23.0%)	
ICU mortality, n (%)	672 (25.7%)	- - -	- - -	- - -	- - -
Hospital mortality, n (%)	759 (29.0%)	121 (6.2%)	- - -	- - -	- - -

AIDS: Acquired Immunodeficiency Syndrome; APACHE: Acute Physiology and Chronic Health Evaluation; CHF: Congestive Heart Failure; COPD: Chronic Obstructive Pulmonary Disease; CRF: Chronic Renal Failure; DM: Diabetes Mellitus; ICU: Intensive Care Unit; MV: Mechanical Ventilation; SH: Systemic Hypertension.

**Table 2:** Physiotherapeutic and nutritional evaluation at the post-ICU outpatient clinic.

Physiotherapeutic evaluation n=591 (with exception of spirometry)		Nutritional evaluation n=542	
**MaxIP, mmHg**		**Current feeding route**	
<{-20}	5.4%	Oral	94.6%
{-20} to {-40}	8.7%	Enteral/gastric tube	2.6%
>{-40}	85.9%	Gastrostomy/Jejunostomy	2.2%
**Muscular strength** (MRC scale)		Oral + Enteral	0.6%
All limbs 4 or 5 (“normal”)	68.2%	**% Weight loss during hospitalization**	
Hemiparesis/hemiplegia	4.9%	0	23.5%
Severe paraparesis or paraplegia	1.6%	0.1-5%	7.5%
Moderate tetraparesis	4.7%	5.1-10%	17.7%
Severe tetraparesis or tetraplegia	1.0%	10.1-20%	31.4%
**Spirometry**, n=427		20.1-30%	12.8%
Normal	51.0%	>30%	7.1%
Restrictive	11.2%	**% Weight gain between discharge from hospital and outpatient consultation**	
Obstructive	37.8%		
**Dyspnea on physical exertion**, Borg scale		0	34.4%
0-1	58.5%	0.1-5%	11.4%
2-4	16.9%	5.1-10%	18.5%
5-8	15.3%	10.1-20%	22.5%
9-10	9.3%	20.1-30%	9.2%
		>30%	4.0%

ICU: Intensive Care Unit; mmHg: Millimeters of mercury; MaxIP: Maximum Inspiratory Pressure; MRC: Medical Research Council; BMI: Body Mass Index.

**Table 3:** Psychological evaluation at the Post-ICU Outpatient Clinic (n=628).

Psychological problems (%) No: 56.9% Yes: 43.1%					
**HADS**	Mean ± SD	0-9		10-20	>20
Anxiety	5.4 ± 4.83	70.8%		29.2%	- - -
Depression	4.7 ± 4.83	77.4%		22.6%	- - -
**IES-R**					
PTSD	9.2 ± 11.81	69.9%		13.9%	16.2%
**Memories of events in the ICU, %**					
No			15.6%		
Yes			84.4%		
Real events			39.1%		
Illusion memories			5.5%		
Combination (illusions and real events)			39.8%		

ICU: Intensive Care Unit; HADS: Hospital Anxiety and Depression Scale; IES-R: Impact of Event Scale-Revised; PTSD: Post-Traumatic Stress Disorder. The incidence of psychological problems also includes patients with more than one condition (e.g., anxiety + depression).

**Supplementary Table:** Comparison of admitted ICU patients to ICU surviving patients.

	ICU Total	ICU Survivors	*p*-value Total *vs*. Survivors
n	2617	1945	- - -
Male, %	1639 (62.6%)	1227 (63.1%)	
Age, years, median ± SD	46.0 ± 19.83	44.0 ± 19.63	<0.001
Age, years, n (%)	- - -	- - -	
<40	1081 (41.3%)	897 (46.1%)	
41-60	803 (30.7%)	567 (29.2%)	
61-75	524 (20.0%)	350 (18.0%)	
>75	209 (8.0%)	131 (6.7%)	
Reason for hospitalization, n (%)	- - -	- - -	- - -
Trauma	963 (36.8%)	761 (39.1%)	0.120
Clinical	916 (35.0%)	570 (29.3%)	<0.001
Elective surgical	429 (16.4%)	393 (20.2%)	0.001
Non-traumatic urgency surgical	309 (11.8%)	221 (11.4%)	0.711
APACHE II, median ± SD	22.0 ± 8.66	20.0 ± 7.77	<0.001
APACHE II, n (%)	- - -	- - -	
1-5	39 (1.5%)	38 (2.0%)	
6-10	188 (7.2%)	174 (8.9%)	
11-20	882 (33.7%)	788 (40.5%)	
21-30	1055 (40.3%)	760 (39.1%)	
>30	453 (17.3%)	185 (9.5%)	
Comorbid conditions, n (%)	- - -	- - -	- - -
Any	1633 (62.4%)	1157 (59.5%)	0.05
None	984 (37.6%)	788 (40.5%)	
AIDS	49 (1.9%)	32 (1.6%)	0.518
Recent/current cancer	248 (10.7%)	225 (11.6%)	0.363
DM	237 (9.1%)	175 (9.0%)	0.949
Illegal drugs	64 (2.4%)	54 (2.8%)	0.453
COPD	175 (6.7%)	87 (4.5%)	0.002
Alcoholism	243 (9.3%)	210 (10.8%)	0.104
SH	589 (22.5%)	486 (25.0%)	0.053
CHF	162 (6.2%)	126 (6.5%)	0.726
CRF	61 (2.3%)	41 (2.1%)	0.725
Moderate/severe obesity	170 (6.5%)	103 (5.3%)	0.104
MV use, n (%)	2269 (86.7%)	1610 (82.8%)	<0.001
Time on MV, days, n (%)	- - -	- - -	- - -
0	348 (13.3%)	335 (17.2%)	<0.001
1-5	1112 (42.5%)	811 (41.7%)	
6-10	487 (18.6%)	335 (17.2%)	
>10	670 (25.6%)	464 (23.9%)	
ICU mortality, %	672 (25.7%)	- - -	- - -
Hospital mortality, n (%)	759 (29.0%)	121 (6.2%)	- - -

AIDS: Acquired Immunodeficiency Syndrome; APACHE: Acute Physiology and Chronic Health Evaluation; CHF: Congestive Heart Failure; COPD: Chronic Obstructive Pulmonary Disease; CRF: Chronic Renal Failure; DM: Diabetes Mellitus; ICU: Intensive Care Unit; MV: Mechanical Ventilation; SH: Systemic Hypertension.
